# Crystal structure of ethyl 1′,1′′-dimethyl-2′′,3-dioxo-3*H*-di­spiro­[benzo[*b*]thio­phene-2,3′-pyrrolidine-2′,3′′-indoline]-4′-carboxyl­ate

**DOI:** 10.1107/S2056989015002042

**Published:** 2015-02-04

**Authors:** M. P. Savithri, M. Suresh, R. Raghunathan, R. Raja, A. SubbiahPandi

**Affiliations:** a Department of Physics, Queen Mary’s College (Autonomous), Chennai 600 004, India; b Department of Organic Chemistry, University of Madras, Guindy Campus, Chennai 600 025, India; c Department of Physics, Presidency College (Autonomous), Chennai 600 005, India

**Keywords:** crystal structure, di­spiro, benzo­thio­phene, pyrrolidine, indole, C—H⋯O hydrogen bonds.

## Abstract

In the title compound, C_23_H_22_N_2_O_4_S, the pyrrolidine ring has an envelope conformation with the spiro C atom, shared with the indoline ring system, as the flap. The mean planes of the benzo­thio­phene and indoline ring systems are inclined to the mean plane of the pyrrolidine ring by 88.81 (8) and 79.48 (8)°, respectively, and to each other by 68.12 (5)°. In the crystal, mol­ecules are linked *via* C—H⋯O hydrogen bonds, forming chains propagating along [001].

## Related literature   

For various biological activities of indole derivatives, see: Harris & Uhle (1960 ▸); Ho *et al.* (1986 ▸); Stevenson *et al.* (2000 ▸). For the crystal structures of two very similar compounds, see: Savithri *et al.* (2014 ▸).
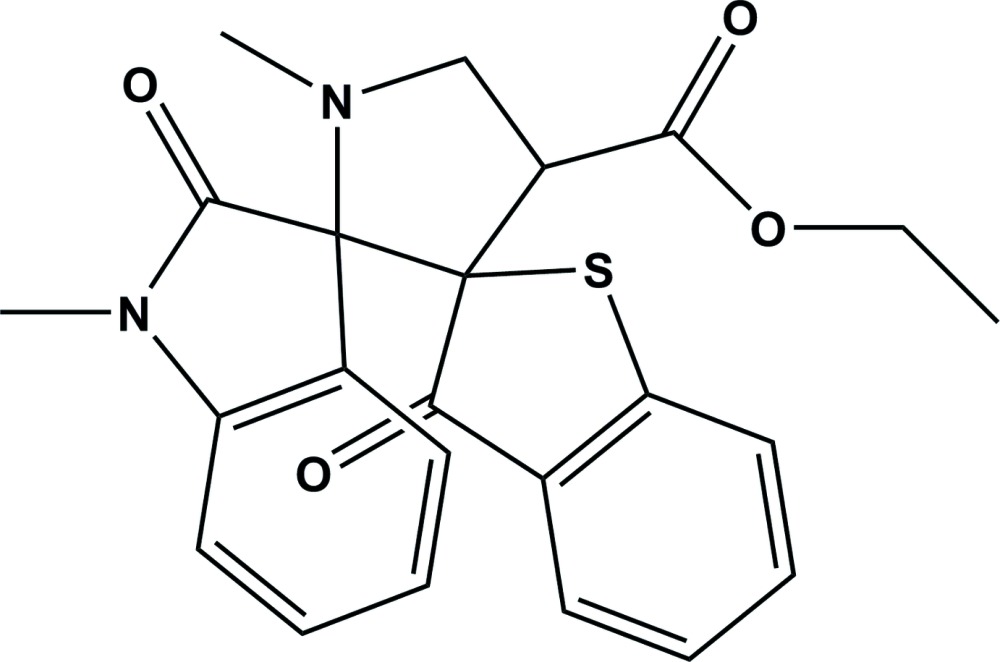


## Experimental   

### Crystal data  

C_23_H_22_N_2_O_4_S
*M* *_r_* = 422.49
Monoclinic, 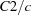

*a* = 23.7049 (11) Å
*b* = 8.2632 (3) Å
*c* = 22.1003 (8) Å
β = 102.337 (2)°
*V* = 4229.0 (3) Å^3^
*Z* = 8
Mo *K*α radiation
μ = 0.19 mm^−1^
*T* = 293 K
0.35 × 0.30 × 0.30 mm

### Data collection  

Bruker Kappa APEXII CCD diffractometer
Absorption correction: multi-scan (*SADABS*; Bruker, 2004 ▸) *T*_min_ = 0.896, *T*_max_ = 0.910
22087 measured reflections
4621 independent reflections
3869 reflections with *I* > 2σ(*I*)
*R*_int_ = 0.029

### Refinement  

*R*[*F* ^2^ > 2σ(*F* ^2^)] = 0.038
*wR*(*F* ^2^) = 0.105
*S* = 1.03
4618 reflections
275 parameters
H-atom parameters constrained
Δρ_max_ = 0.40 e Å^−3^
Δρ_min_ = −0.20 e Å^−3^

### 

Data collection: *APEX2* (Bruker, 2004 ▸); cell refinement: *APEX2* and *SAINT* (Bruker, 2004 ▸); data reduction: *SAINT* and *XPREP* (Bruker, 2004 ▸); program(s) used to solve structure: *SHELXS97* (Sheldrick, 2008 ▸); program(s) used to refine structure: *SHELXL97* (Sheldrick, 2008 ▸, 2015 ▸); molecular graphics: *ORTEP-3 for Windows* (Farrugia, 2012 ▸); software used to prepare material for publication: *SHELXL97* and *PLATON* (Spek, 2009 ▸).

## Supplementary Material

Crystal structure: contains datablock(s) global, I. DOI: 10.1107/S2056989015002042/su5065sup1.cif

Structure factors: contains datablock(s) I. DOI: 10.1107/S2056989015002042/su5065Isup2.hkl

**Fig. 1.**
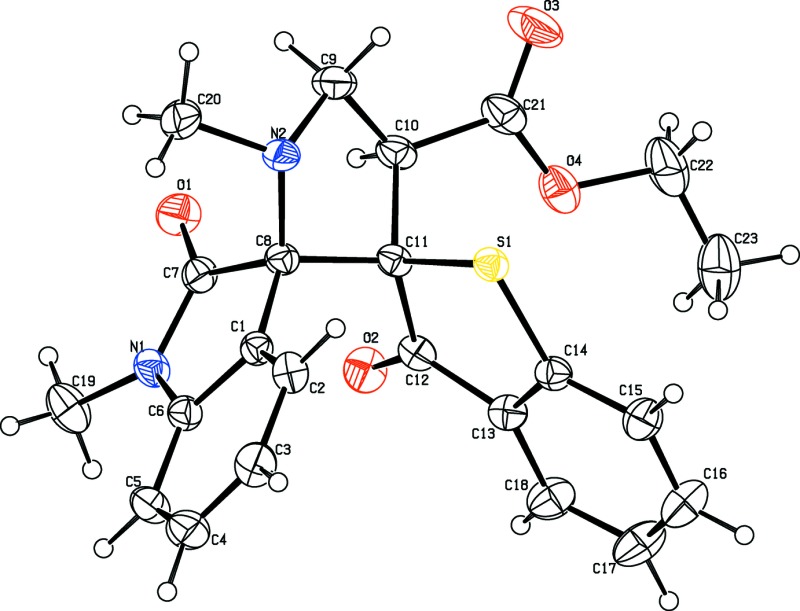
The molecular structure of the title compound with the atom numbering scheme. Displacement ellipsoids are drawn at the 30% probability level.

**Fig. 2.**
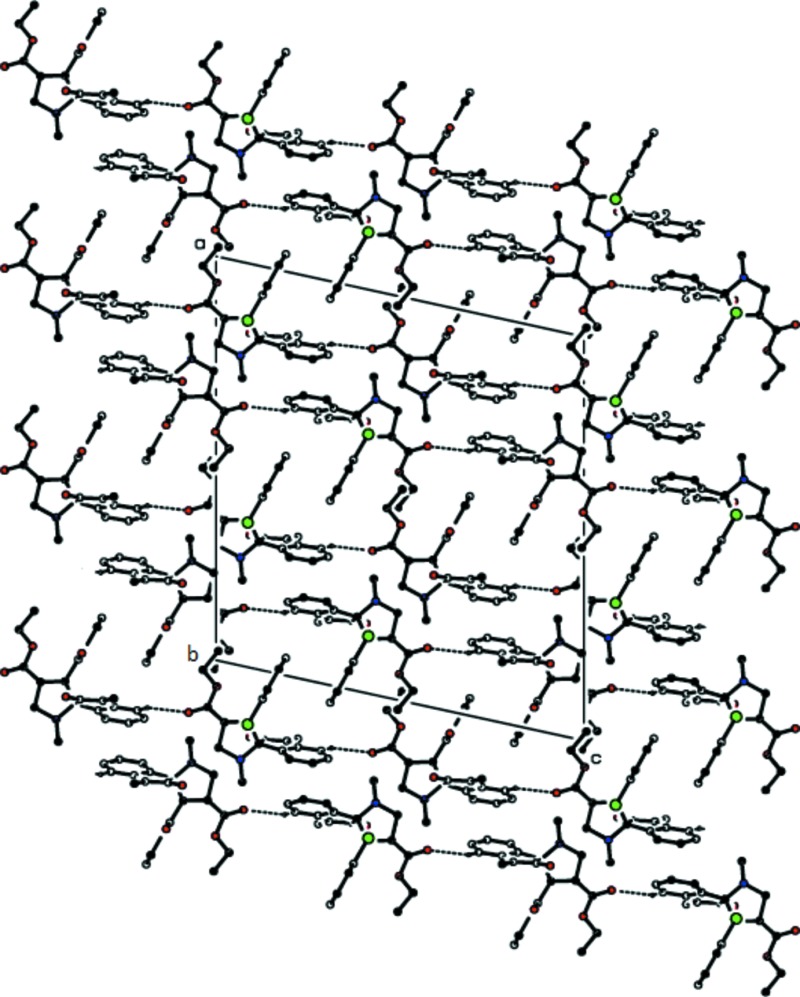
The crystal packing of the title compound viewed along the b axis. Dashed lines shows the intermolecular C—H···O hydrogen bonds. H atoms not involved in hydrogen bonding have been omitted for clarity.

. DOI: 10.1107/S2056989015002042/su5065fig1.tif
The mol­ecular structure of the title compound with the atom numbering scheme. Displacement ellipsoids are drawn at the 30% probability level.

b . DOI: 10.1107/S2056989015002042/su5065fig2.tif
The crystal packing of the title compound viewed along the *b* axis. Dashed lines shows the inter­molecular C—H⋯O hydrogen bonds. H atoms not involved in hydrogen bonding have been omitted for clarity.

CCDC reference: 1046459

Additional supporting information:  crystallographic information; 3D view; checkCIF report

## Figures and Tables

**Table 1:** Hydrogen-bond geometry (, )

*D*H*A*	*D*H	H*A*	*D* *A*	*D*H*A*
C5H5O3^i^	0.93	2.46	3.212(2)	138

Symmetry code: (i) 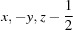.

## supplementary crystallographic information

### S1. Comment

Indole compounds can be used as bioactive drugs (Stevenson *et al.*, 2000). Indole derivatives exhibit antiallergic, central nervous system depressant and muscle relaxant properties (Harris & Uhle, 1960; Ho *et al.*, 1986). In view of this biological importance, the crystal structure of the title compound was determined and the results are presented here.

The X-ray analysis confirms the molecular structure and atom connectivity as illustrated in Fig. 1. The geometric parameters of the title molecule agrees well with those reported for similar structures (Savithri *et al.*, 2014).

The five membered ring (N1/C1/C6-C8) in the indole moiety adopts an envelope conformation with the C8 as the flap atom [puckering parameters q_2_ = 0.0888 (2)Å and φ_2_ = 284.2 (1)°] and the pyrrolidine ring (N2/C8-C11) exhibits a twisted conformation [puckering parameters, q_2_ = 0.4626 (2)Å and φ_2_ = 233.4 (2)°]. The bond length C12-O2 = 1.211 Å indicates a keto group in the benzothiophene. The sum of angles at N2 of the pyrrolidine ring (339°) is in accordance with *sp*^3^ hybridization and the sum of angles at N1 of the indole moiety (359°) is in accordance with *sp*^2^ hybridization.

The pyrrolidine ring (N2/C8-C11) is perpendicular with benzothiophene (C11-C18/S1) oriented at a dihedral angle of 88.81 (8)° and is oriented with indole ring (N1/C1-C8) at a dihedral angle of 79.48 (8)°. The thiophene ring (C11-C14/S1) makes dihedral angles of 87.98 (8)° and 67.14 (6)° with pyrrolidine (N2/C8-C11) and indole (N1/C1-C8) rings, respectively.

In the crystal, hydrogen-bonded chains running along [001] are generated by connecting neighbouring molecules via C-H···O hydrogen bonds (Table 1 and Fig. 2).

### S2. Experimental

(*E*)-ethyl 2-(3-oxobenzo[b]thiophen-2(3H)-ylidene) acetate (1.0 mmol), N-methyl isatin (1.1 mmol) and sarcosine (1.1 mmol) were refluxed in methanol (20ml) until completion of the reaction monitored by TLC analysis. After completion of the reaction the solvent was evaporated under reduced pressure. The crude reaction mixture was dissolved in dichloromethane (2 × 50 ml) and washed with water followed by brine solution. The organic layer was separated and dried over sodium sulfate. After filtration the organic solvent was evaporated under reduced pressure. The product was separated by column chromatography using hexane and ethyl acetate (9:1) as an eluent to give a colourless solid. The product was dissolved in chloroform (3 ml) and heated for 2 min. The resulting solution was subjected to crystallization by slow evaporation of the solvent giving in single crystals suitable for X-ray crystallographic studies.

### S3. Refinement

All H atoms were fixed geometrically and allowed to ride on their parent C atom: C—H = 0.93–0.98 Å with *U*_iso_(H) = 1.5*U*_eq_(C) for methyl H atoms and = 1.2*U*_eq_(C) for other H atoms.

### Crystal data

**Table:**

C_23_H_22_N_2_O_4_S	*F*(000) = 1776
*M**_r_* = 422.49	*D*_x_ = 1.327 Mg m^−^^3^
Monoclinic, *C*2/*c*	Mo *K*α radiation, λ = 0.71073 Å
Hall symbol: -C 2yc	Cell parameters from 4634 reflections
*a* = 23.7049 (11) Å	θ = 2.3–27.0°
*b* = 8.2632 (3) Å	µ = 0.19 mm^−^^1^
*c* = 22.1003 (8) Å	*T* = 293 K
β = 102.337 (2)°	Block, colourless
*V* = 4229.0 (3) Å^3^	0.35 × 0.30 × 0.30 mm
*Z* = 8	

### Data collection

**Table:**

Bruker Kappa APEXII CCD diffractometer	4621 independent reflections
Radiation source: fine-focus sealed tube	3869 reflections with *I* > 2σ(*I*)
Graphite monochromator	*R*_int_ = 0.029
ω and φ scans	θ_max_ = 27.0°, θ_min_ = 2.3°
Absorption correction: multi-scan (*SADABS*; Bruker, 2004)	*h* = −30→30
*T*_min_ = 0.896, *T*_max_ = 0.910	*k* = −10→8
22087 measured reflections	*l* = −22→28

### Refinement

**Table:**

Refinement on *F*^2^	Secondary atom site location: difference Fourier map
Least-squares matrix: full	Hydrogen site location: inferred from neighbouring sites
*R*[*F*^2^ > 2σ(*F*^2^)] = 0.038	H-atom parameters constrained
*wR*(*F*^2^) = 0.105	*w* = 1/[σ^2^(*F*_o_^2^) + (0.0502*P*)^2^ + 2.8328*P*] where *P* = (*F*_o_^2^ + 2*F*_c_^2^)/3
*S* = 1.03	(Δ/σ)_max_ < 0.001
4618 reflections	Δρ_max_ = 0.40 e Å^−^^3^
275 parameters	Δρ_min_ = −0.20 e Å^−^^3^
0 restraints	Extinction correction: *SHELXL97* (Sheldrick, 2008, 2015), Fc^*^=kFc[1+0.001xFc^2^λ^3^/sin(2θ)]^-1/4^
Primary atom site location: structure-invariant direct methods	Extinction coefficient: 0.0025 (2)

### Special details

**Table:**

Geometry. All esds (except the esd in the dihedral angle between two l.s. planes) are estimated using the full covariance matrix. The cell esds are taken into account individually in the estimation of esds in distances, angles and torsion angles; correlations between esds in cell parameters are only used when they are defined by crystal symmetry. An approximate (isotropic) treatment of cell esds is used for estimating esds involving l.s. planes.
Refinement. Refinement of F^2^ against ALL reflections. The weighted R-factor wR and goodness of fit S are based on F^2^, conventional R-factors R are based on F, with F set to zero for negative F^2^. The threshold expression of F^2^ > 2sigma(F^2^) is used only for calculating R-factors(gt) etc. and is not relevant to the choice of reflections for refinement. R-factors based on F^2^ are statistically about twice as large as those based on F, and R- factors based on ALL data will be even larger.

### Fractional atomic coordinates and isotropic or equivalent isotropic displacement parameters (Å^2^)

**Table:**

	*x*	*y*	*z*	*U*_iso_*/*U*_eq_	
S1	0.141873 (15)	0.31779 (4)	0.413751 (16)	0.03357 (12)	
C11	0.13418 (6)	0.09712 (17)	0.41066 (6)	0.0316 (3)	
N1	0.16154 (6)	−0.18548 (15)	0.30761 (6)	0.0390 (3)	
C1	0.18787 (6)	0.08191 (18)	0.32013 (6)	0.0319 (3)	
O2	0.05531 (5)	−0.07835 (14)	0.36475 (6)	0.0499 (3)	
O1	0.17197 (6)	−0.27090 (14)	0.40812 (6)	0.0515 (3)	
C8	0.18360 (6)	0.02047 (17)	0.38341 (6)	0.0318 (3)	
C12	0.07265 (6)	0.05908 (19)	0.37426 (7)	0.0350 (3)	
C14	0.07062 (6)	0.34826 (19)	0.37340 (7)	0.0352 (3)	
C7	0.17122 (6)	−0.16405 (18)	0.37017 (7)	0.0364 (3)	
N2	0.23289 (5)	0.05295 (16)	0.43401 (6)	0.0384 (3)	
C13	0.03980 (6)	0.2070 (2)	0.35486 (7)	0.0380 (3)	
C6	0.17075 (6)	−0.04172 (19)	0.27721 (7)	0.0356 (3)	
C2	0.20526 (7)	0.2287 (2)	0.30060 (8)	0.0395 (4)	
H2	0.2187	0.3105	0.3290	0.047*	
O4	0.06476 (6)	0.13849 (16)	0.49836 (6)	0.0556 (3)	
C10	0.14909 (7)	0.0263 (2)	0.47706 (7)	0.0405 (4)	
H10	0.1360	−0.0865	0.4747	0.049*	
C9	0.21520 (8)	0.0253 (2)	0.49324 (8)	0.0495 (4)	
H9A	0.2298	0.1104	0.5226	0.059*	
H9B	0.2296	−0.0780	0.5110	0.059*	
C15	0.04484 (7)	0.4995 (2)	0.36049 (8)	0.0471 (4)	
H15	0.0654	0.5942	0.3726	0.056*	
C20	0.28698 (7)	−0.0239 (2)	0.42869 (9)	0.0541 (5)	
H20A	0.2826	−0.1394	0.4290	0.081*	
H20B	0.3170	0.0083	0.4630	0.081*	
H20C	0.2969	0.0088	0.3906	0.081*	
C18	−0.01766 (8)	0.2148 (2)	0.32301 (9)	0.0539 (5)	
H18	−0.0384	0.1206	0.3105	0.065*	
C21	0.12051 (9)	0.1125 (2)	0.52268 (7)	0.0498 (4)	
C5	0.16713 (7)	−0.0205 (2)	0.21453 (7)	0.0474 (4)	
H5	0.1547	−0.1032	0.1863	0.057*	
C3	0.20234 (8)	0.2522 (2)	0.23745 (8)	0.0489 (4)	
H3	0.2136	0.3508	0.2236	0.059*	
O3	0.14426 (8)	0.1504 (2)	0.57445 (6)	0.0794 (5)	
C4	0.18291 (8)	0.1304 (3)	0.19547 (8)	0.0530 (5)	
H4	0.1803	0.1495	0.1535	0.064*	
C19	0.15024 (10)	−0.3419 (2)	0.27783 (10)	0.0598 (5)	
H19A	0.1551	−0.4252	0.3088	0.090*	
H19B	0.1767	−0.3596	0.2511	0.090*	
H19C	0.1114	−0.3445	0.2538	0.090*	
C16	−0.01201 (9)	0.5045 (3)	0.32919 (11)	0.0646 (5)	
H16	−0.0300	0.6043	0.3204	0.078*	
C17	−0.04313 (8)	0.3642 (3)	0.31035 (11)	0.0686 (6)	
H17	−0.0814	0.3713	0.2891	0.082*	
C22	0.03086 (12)	0.2257 (3)	0.53552 (11)	0.0763 (7)	
H22A	0.0519	0.3201	0.5544	0.092*	
H22B	0.0230	0.1565	0.5682	0.092*	
C23	−0.02388 (11)	0.2757 (3)	0.49378 (13)	0.0819 (7)	
H23A	−0.0156	0.3458	0.4621	0.123*	
H23B	−0.0475	0.3320	0.5172	0.123*	
H23C	−0.0440	0.1815	0.4749	0.123*	

### Atomic displacement parameters (Å^2^)

**Table:**

	*U*^11^	*U*^22^	*U*^33^	*U*^12^	*U*^13^	*U*^23^
S1	0.0366 (2)	0.0301 (2)	0.03283 (19)	−0.00394 (14)	0.00468 (14)	−0.00350 (15)
C11	0.0373 (7)	0.0291 (7)	0.0280 (6)	−0.0048 (6)	0.0063 (5)	−0.0004 (6)
N1	0.0449 (7)	0.0305 (7)	0.0405 (7)	−0.0051 (5)	0.0066 (5)	−0.0084 (6)
C1	0.0295 (7)	0.0325 (8)	0.0336 (7)	0.0001 (5)	0.0066 (5)	−0.0022 (6)
O2	0.0461 (6)	0.0378 (7)	0.0646 (8)	−0.0129 (5)	0.0090 (6)	−0.0061 (6)
O1	0.0686 (8)	0.0314 (6)	0.0514 (7)	−0.0028 (5)	0.0059 (6)	0.0072 (6)
C8	0.0346 (7)	0.0279 (7)	0.0309 (7)	−0.0026 (6)	0.0028 (5)	−0.0017 (6)
C12	0.0363 (7)	0.0380 (8)	0.0319 (7)	−0.0083 (6)	0.0103 (6)	−0.0018 (6)
C14	0.0354 (7)	0.0394 (8)	0.0314 (7)	−0.0017 (6)	0.0087 (6)	0.0023 (6)
C7	0.0374 (8)	0.0297 (8)	0.0402 (8)	−0.0012 (6)	0.0042 (6)	−0.0028 (7)
N2	0.0368 (7)	0.0393 (7)	0.0344 (6)	−0.0001 (5)	−0.0027 (5)	−0.0052 (6)
C13	0.0351 (7)	0.0424 (9)	0.0363 (8)	−0.0039 (6)	0.0074 (6)	0.0016 (7)
C6	0.0324 (7)	0.0380 (8)	0.0357 (7)	−0.0004 (6)	0.0056 (6)	−0.0049 (6)
C2	0.0405 (8)	0.0361 (8)	0.0447 (9)	−0.0032 (6)	0.0153 (7)	−0.0022 (7)
O4	0.0676 (8)	0.0583 (8)	0.0484 (7)	−0.0018 (6)	0.0292 (6)	−0.0051 (6)
C10	0.0561 (9)	0.0360 (8)	0.0286 (7)	−0.0033 (7)	0.0071 (6)	0.0036 (6)
C9	0.0574 (10)	0.0516 (10)	0.0339 (8)	0.0029 (8)	−0.0028 (7)	0.0021 (8)
C15	0.0486 (9)	0.0402 (9)	0.0527 (10)	0.0024 (7)	0.0116 (8)	0.0048 (8)
C20	0.0412 (9)	0.0573 (11)	0.0574 (11)	0.0070 (8)	−0.0038 (8)	−0.0107 (9)
C18	0.0388 (9)	0.0560 (11)	0.0624 (11)	−0.0086 (8)	0.0010 (8)	0.0020 (9)
C21	0.0757 (12)	0.0444 (10)	0.0326 (8)	−0.0061 (9)	0.0187 (8)	0.0056 (7)
C5	0.0516 (9)	0.0556 (11)	0.0341 (8)	0.0001 (8)	0.0075 (7)	−0.0095 (8)
C3	0.0518 (10)	0.0489 (10)	0.0511 (10)	0.0011 (8)	0.0223 (8)	0.0104 (8)
O3	0.1083 (13)	0.0999 (13)	0.0299 (6)	0.0017 (10)	0.0146 (7)	−0.0060 (7)
C4	0.0585 (11)	0.0672 (12)	0.0359 (8)	0.0068 (9)	0.0160 (7)	0.0067 (9)
C19	0.0756 (13)	0.0414 (10)	0.0633 (12)	−0.0176 (9)	0.0168 (10)	−0.0216 (9)
C16	0.0524 (11)	0.0571 (12)	0.0803 (14)	0.0142 (9)	0.0051 (10)	0.0150 (11)
C17	0.0396 (9)	0.0744 (14)	0.0835 (15)	0.0060 (10)	−0.0054 (9)	0.0138 (12)
C22	0.1001 (18)	0.0769 (16)	0.0680 (14)	0.0025 (13)	0.0542 (14)	−0.0079 (12)
C23	0.0883 (17)	0.0679 (15)	0.106 (2)	0.0066 (13)	0.0587 (16)	−0.0015 (14)

### Geometric parameters (Å, º)

**Table:**

S1—C14	1.7516 (15)	C10—H10	0.9800
S1—C11	1.8324 (15)	C9—H9A	0.9700
C11—C12	1.540 (2)	C9—H9B	0.9700
C11—C10	1.5487 (19)	C15—C16	1.378 (3)
C11—C8	1.561 (2)	C15—H15	0.9300
N1—C7	1.364 (2)	C20—H20A	0.9600
N1—C6	1.405 (2)	C20—H20B	0.9600
N1—C19	1.449 (2)	C20—H20C	0.9600
C1—C2	1.380 (2)	C18—C17	1.377 (3)
C1—C6	1.394 (2)	C18—H18	0.9300
C1—C8	1.511 (2)	C21—O3	1.204 (2)
O2—C12	1.2107 (18)	C5—C4	1.393 (3)
O1—C7	1.2153 (19)	C5—H5	0.9300
C8—N2	1.4592 (18)	C3—C4	1.379 (3)
C8—C7	1.568 (2)	C3—H3	0.9300
C12—C13	1.464 (2)	C4—H4	0.9300
C14—C13	1.391 (2)	C19—H19A	0.9600
C14—C15	1.394 (2)	C19—H19B	0.9600
N2—C20	1.458 (2)	C19—H19C	0.9600
N2—C9	1.475 (2)	C16—C17	1.389 (3)
C13—C18	1.395 (2)	C16—H16	0.9300
C6—C5	1.381 (2)	C17—H17	0.9300
C2—C3	1.396 (2)	C22—C23	1.481 (4)
C2—H2	0.9300	C22—H22A	0.9700
O4—C21	1.333 (2)	C22—H22B	0.9700
O4—C22	1.457 (2)	C23—H23A	0.9600
C10—C21	1.508 (2)	C23—H23B	0.9600
C10—C9	1.531 (2)	C23—H23C	0.9600
			
C14—S1—C11	92.67 (7)	C10—C9—H9B	110.7
C12—C11—C10	114.63 (12)	H9A—C9—H9B	108.8
C12—C11—C8	114.87 (11)	C16—C15—C14	117.94 (17)
C10—C11—C8	100.04 (11)	C16—C15—H15	121.0
C12—C11—S1	107.34 (10)	C14—C15—H15	121.0
C10—C11—S1	109.86 (10)	N2—C20—H20A	109.5
C8—C11—S1	109.95 (9)	N2—C20—H20B	109.5
C7—N1—C6	111.44 (12)	H20A—C20—H20B	109.5
C7—N1—C19	123.43 (14)	N2—C20—H20C	109.5
C6—N1—C19	124.72 (14)	H20A—C20—H20C	109.5
C2—C1—C6	119.63 (14)	H20B—C20—H20C	109.5
C2—C1—C8	131.94 (13)	C17—C18—C13	118.84 (17)
C6—C1—C8	108.43 (12)	C17—C18—H18	120.6
N2—C8—C1	116.51 (12)	C13—C18—H18	120.6
N2—C8—C11	100.00 (11)	O3—C21—O4	124.29 (18)
C1—C8—C11	115.14 (11)	O3—C21—C10	124.94 (19)
N2—C8—C7	114.14 (12)	O4—C21—C10	110.76 (14)
C1—C8—C7	101.59 (11)	C6—C5—C4	117.01 (16)
C11—C8—C7	109.90 (11)	C6—C5—H5	121.5
O2—C12—C13	126.37 (14)	C4—C5—H5	121.5
O2—C12—C11	122.04 (14)	C4—C3—C2	120.62 (17)
C13—C12—C11	111.58 (12)	C4—C3—H3	119.7
C13—C14—C15	120.82 (14)	C2—C3—H3	119.7
C13—C14—S1	114.70 (12)	C3—C4—C5	121.49 (16)
C15—C14—S1	124.48 (13)	C3—C4—H4	119.3
O1—C7—N1	125.43 (14)	C5—C4—H4	119.3
O1—C7—C8	127.09 (14)	N1—C19—H19A	109.5
N1—C7—C8	107.43 (12)	N1—C19—H19B	109.5
C20—N2—C8	115.60 (12)	H19A—C19—H19B	109.5
C20—N2—C9	115.19 (13)	N1—C19—H19C	109.5
C8—N2—C9	108.58 (12)	H19A—C19—H19C	109.5
C14—C13—C18	120.33 (15)	H19B—C19—H19C	109.5
C14—C13—C12	113.66 (13)	C15—C16—C17	121.70 (18)
C18—C13—C12	125.96 (15)	C15—C16—H16	119.2
C5—C6—C1	122.43 (15)	C17—C16—H16	119.2
C5—C6—N1	127.21 (15)	C18—C17—C16	120.38 (17)
C1—C6—N1	110.28 (13)	C18—C17—H17	119.8
C1—C2—C3	118.71 (15)	C16—C17—H17	119.8
C1—C2—H2	120.6	O4—C22—C23	107.59 (18)
C3—C2—H2	120.6	O4—C22—H22A	110.2
C21—O4—C22	118.09 (16)	C23—C22—H22A	110.2
C21—C10—C9	115.47 (14)	O4—C22—H22B	110.2
C21—C10—C11	114.26 (13)	C23—C22—H22B	110.2
C9—C10—C11	103.79 (12)	H22A—C22—H22B	108.5
C21—C10—H10	107.6	C22—C23—H23A	109.5
C9—C10—H10	107.6	C22—C23—H23B	109.5
C11—C10—H10	107.6	H23A—C23—H23B	109.5
N2—C9—C10	105.23 (12)	C22—C23—H23C	109.5
N2—C9—H9A	110.7	H23A—C23—H23C	109.5
C10—C9—H9A	110.7	H23B—C23—H23C	109.5
N2—C9—H9B	110.7		
			
C14—S1—C11—C12	1.58 (10)	S1—C14—C13—C12	1.56 (17)
C14—S1—C11—C10	−123.66 (11)	O2—C12—C13—C14	178.78 (15)
C14—S1—C11—C8	127.17 (10)	C11—C12—C13—C14	−0.26 (18)
C2—C1—C8—N2	−45.8 (2)	O2—C12—C13—C18	1.3 (3)
C6—C1—C8—N2	133.48 (13)	C11—C12—C13—C18	−177.73 (15)
C2—C1—C8—C11	70.9 (2)	C2—C1—C6—C5	−3.9 (2)
C6—C1—C8—C11	−109.88 (13)	C8—C1—C6—C5	176.69 (14)
C2—C1—C8—C7	−170.46 (15)	C2—C1—C6—N1	172.93 (13)
C6—C1—C8—C7	8.80 (14)	C8—C1—C6—N1	−6.44 (16)
C12—C11—C8—N2	−169.56 (12)	C7—N1—C6—C5	177.34 (15)
C10—C11—C8—N2	−46.29 (13)	C19—N1—C6—C5	4.5 (3)
S1—C11—C8—N2	69.26 (11)	C7—N1—C6—C1	0.65 (17)
C12—C11—C8—C1	64.75 (16)	C19—N1—C6—C1	−172.18 (15)
C10—C11—C8—C1	−171.98 (12)	C6—C1—C2—C3	3.3 (2)
S1—C11—C8—C1	−56.43 (14)	C8—C1—C2—C3	−177.51 (15)
C12—C11—C8—C7	−49.19 (16)	C12—C11—C10—C21	−73.44 (17)
C10—C11—C8—C7	74.08 (13)	C8—C11—C10—C21	163.12 (13)
S1—C11—C8—C7	−170.37 (9)	S1—C11—C10—C21	47.50 (16)
C10—C11—C12—O2	−57.80 (19)	C12—C11—C10—C9	159.94 (13)
C8—C11—C12—O2	57.28 (19)	C8—C11—C10—C9	36.51 (14)
S1—C11—C12—O2	179.88 (12)	S1—C11—C10—C9	−79.11 (13)
C10—C11—C12—C13	121.29 (14)	C20—N2—C9—C10	−148.67 (14)
C8—C11—C12—C13	−123.63 (13)	C8—N2—C9—C10	−17.23 (17)
S1—C11—C12—C13	−1.03 (14)	C21—C10—C9—N2	−139.30 (14)
C11—S1—C14—C13	−1.87 (12)	C11—C10—C9—N2	−13.45 (17)
C11—S1—C14—C15	177.39 (14)	C13—C14—C15—C16	0.3 (2)
C6—N1—C7—O1	−172.45 (15)	S1—C14—C15—C16	−178.89 (14)
C19—N1—C7—O1	0.5 (3)	C14—C13—C18—C17	0.0 (3)
C6—N1—C7—C8	5.18 (17)	C12—C13—C18—C17	177.33 (18)
C19—N1—C7—C8	178.12 (15)	C22—O4—C21—O3	3.3 (3)
N2—C8—C7—O1	42.9 (2)	C22—O4—C21—C10	−177.97 (16)
C1—C8—C7—O1	169.17 (15)	C9—C10—C21—O3	−15.2 (3)
C11—C8—C7—O1	−68.47 (19)	C11—C10—C21—O3	−135.47 (19)
N2—C8—C7—N1	−134.66 (13)	C9—C10—C21—O4	166.10 (14)
C1—C8—C7—N1	−8.42 (15)	C11—C10—C21—O4	45.81 (19)
C11—C8—C7—N1	113.95 (13)	C1—C6—C5—C4	1.6 (2)
C1—C8—N2—C20	−63.90 (18)	N1—C6—C5—C4	−174.69 (15)
C11—C8—N2—C20	171.35 (14)	C1—C2—C3—C4	−0.5 (2)
C7—C8—N2—C20	54.10 (18)	C2—C3—C4—C5	−1.8 (3)
C1—C8—N2—C9	164.88 (13)	C6—C5—C4—C3	1.2 (3)
C11—C8—N2—C9	40.13 (14)	C14—C15—C16—C17	−0.5 (3)
C7—C8—N2—C9	−77.11 (16)	C13—C18—C17—C16	−0.2 (3)
C15—C14—C13—C18	−0.1 (2)	C15—C16—C17—C18	0.4 (4)
S1—C14—C13—C18	179.19 (13)	C21—O4—C22—C23	165.63 (18)
C15—C14—C13—C12	−177.72 (14)		

### Hydrogen-bond geometry (Å, º)

**Table:**

*D*—H···*A*	*D*—H	H···*A*	*D*···*A*	*D*—H···*A*
C5—H5···O3^i^	0.93	2.46	3.212 (2)	138

Symmetry code: (i) *x*, −*y*, *z*−1/2.
