# Supplementary figures and images for: Crystal structure of ethyl 1′,1′′-dimethyl-2′′,3-dioxo-3H-di­spiro­[benzo[b]thio­phene-2,3′-pyrrolidine-2′,3′′-indoline]-4′-carboxyl­ate
Source: Acta Crystallogr E Crystallogr Commun. 2015 Feb 4;71(Pt 3):o142. doi: 10.1107/S2056989015002042 (PMC4350758; doi:10.1107/S2056989015002042)

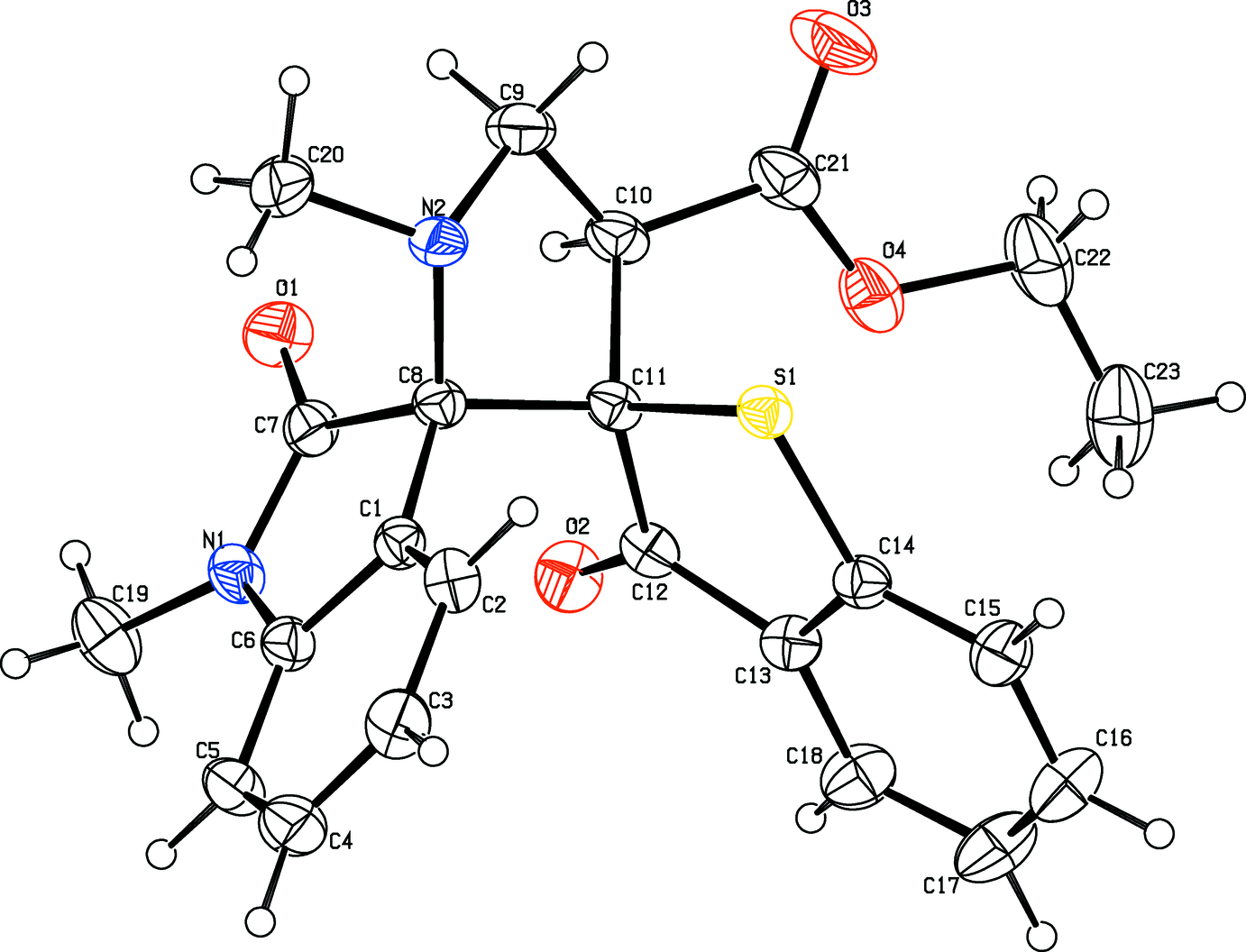

Supplement: Supplementary file 3 [file e-71-0o142-fig1.tif]

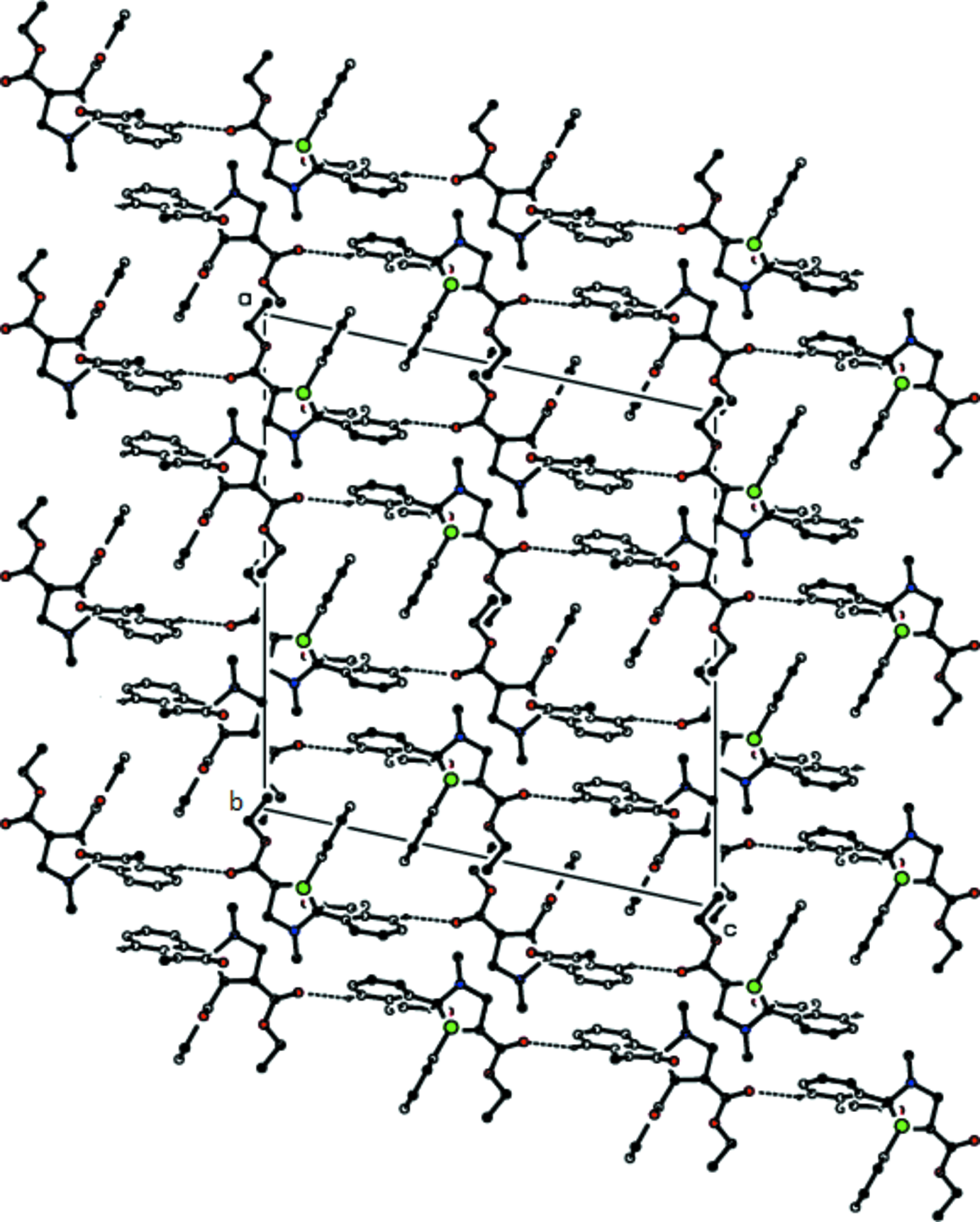

Supplement: Supplementary file 4 [file e-71-0o142-fig2.tif]
